# Antitumoral Drug: Loaded Hybrid Nanocapsules Based on Chitosan with Potential Effects in Breast Cancer Therapy

**DOI:** 10.3390/ijms21165659

**Published:** 2020-08-07

**Authors:** Kheira Zanoune Dellali, Delia Mihaela Rata, Marcel Popa, M’hamed Djennad, Abdallah Ouagued, Daniela Gherghel

**Affiliations:** 1 Laboratory of Structure, Elaboration and Application of Molecular Materials, University Abdelhamid Ibn Badis of Mostaganem, Mostaganem 27000, Algeria; zanounekheira@yahoo.fr (K.Z.D.); mhamed.djennad@univ-mosta.dz (M.D.); 2 Faculty of Technology, University Hassiba Benbouali of Chlef, Chlef BP 151 02000, Algeria; abouagued@yahoo.fr; 3 Department of Natural and Synthetic Polymers, Gheorghe Asachi Technical University of Iasi, Mangeron Bld. no. 73, 700050 Iasi, Romania; 4 Faculty of Medical Dentistry, “Apollonia” University of Iasi, Pacurari Street, No. 11, 700511Iasi, Romania; 5 Academy of Romanian Scientists, Splaiul Independentei Street, No 54, 050094 Bucharest, Romania; 6 Department of Experimental and Applied Biology, NIRDBS-Institute of Biological Research Iasi, Lascar Catargi 47, 700107 Iasi, Romania; daniela_gherghel@yahoo.com

**Keywords:** hybrid nanocapsules, smart polymers, 5-fluorouracil, antitumoral therapy, breast carcinoma

## Abstract

Cancer remains one of the world’s most devastating diseases and is responsible for more than 20% of all deaths. It is defined as uncontrolled proliferation of cells and spreads rapidly to healthy tissue. Controlled drug delivery systems offers great opportunities for the development of new non-invasive strategies for the treatment of cancers. The main advantage of these systems is their capacity to accumulate in tumors via enhanced permeability and retention effects. In the present study, an innovative hybrid drug delivery system based on nanocapsules obtained from the interfacial condensation between chitosan and poly(N-vinyl pyrrolidone-*alt*-itaconic anhydride) and containing both magnetic nanoparticles and an antitumoral drug was developed in order to improve the efficiency of the antitumoral treatment. Using dynamic light scattering, it was observed that the mean diameter of these hybrid nanocapsules was in the range of 43 to 142 nm. SEM confirmed their nanometric size and their well-defined spherical shape. These nanocapsules allowed the encapsulation of an increased amount of 5-fluorouracil and provided controlled drug release. In vitro studies have revealed that these drug-loaded hybrid nanocapsules were able to induce a cytostatic effect on breast carcinoma MCF-7 cell lines (Human Caucasian breast adenocarcinoma - HTB-22) comparable to that of the free drug.

## 1. Introduction

Discovering new cancer treatments is one of the research priorities worldwide and extensive efforts are being made to find a competent treatment strategy, although only limited success has been achieved to date. Classical cancer treatments include chemotherapy, radiotherapy, or their combination, which are generally associated with a series of inconveniences, such as poor bioavailability, high dose requirements, adverse side effects (cytotoxicity, neurotoxicity, nephrotoxicity), low therapeutic indices, development of multiple drug resistance, and non-specific targeting [1,2,3,4]. The application of nanotechnology to oncology presents many benefits [5] and the development of new drug-loaded nanocarriers (nanoparticles, nanocapsules, liposomes, and micelles) can provide great opportunities for cancer treatment because they present several advantages, such as facilitating intratumoral distribution, protecting the active principle from premature degradation, and allowing sustained and controlled release of drugs [1,6,7]. Nanocapsules (NCs) are vesicular systems that have a typical core–shell structure in which active molecules are retained in a reservoir or within a cavity surrounded by a polymer membrane [8]. Polymeric nanocapsules (NCs) can prolong the drug circulation time and delay drug release, can facilitate cellular uptake by the endocytosis mechanism, improve drug targeting, provide controlled drug release [9], and can be loaded with two or more drugs, which may be released in a sequential manner [10]. The shape, size, and surface chemistry of NCs can influence their circulation period and distribution pattern in the body. Thereby, small NCs with a spherical shape and a smooth texture are considered ideal for delivering chemotherapeutic agents to solid tumors, allowing easy transport through the tumor vasculature and into tumor cells [11]. Considerable efforts have been addressed towards the development of multifunctional nanocarriers that deliver anticancer agents directly to diseased tissues via sophisticated targeting strategies [12]. Integration of inorganic nanomaterials (e.g., quantum dots, gold nanoparticles, magnetic nanoparticles, and carbon nanotubes) into various multifunctional nanoparticles has been demonstrated to have potential in cancer therapy [13].

Drug delivery systems based on magnetic nanoparticles (NPs) have attracted increasing attention due to their potential applications in magnetic resonance imaging, photodynamic therapy, and targeted drug delivery [14]. Magnetic NPs consist of magnetic elements such as iron, nickel, cobalt, chromium, manganese, gadolinium, and their chemical compounds, with the most explored magnetic NPs being ferrite nanoparticles [15]. Magnetic NPs have a narrow size distribution; good dispersibility; possess good biocompatibility, low toxicity, and high chemical stability under physiological conditions; and are promising candidates for multifunctional nanocarriers because of their unique and fascinating superparamagnetism [16]. Previous studies have shown that the use of magnetic NPs as heat generators can induce localized cell death [17]. Inspired by these findings, researchers have sought to combine chemotherapy and thermal therapy in order to achieve an efficient cancer treatment [18]. Therefore, the original approach proposed in this study was to develop an innovative hybrid system using NCs based on a natural (chitosan (CS)) and a synthetic polymer (poly(*N*-vinyl pyrrolidone-*alt*-itaconic anhydride)), containing both magnetic NPs and an antitumoral model drug (5-Fluorouracil), in order to improve the efficiency of the antitumoral treatment. Rata et al. [1] have already successfully obtained non-magnetic NCs based on chitosan (CS) and poly(N-vinyl pyrrolidone-*alt*-itaconic anhydride)-poly(NVPAI) by interfacial condensation. The novelty of this system is based on the fact that the synthetic copolymer forms the exterior layer of the polymeric membrane of the NCs. The encapsulation of magnetic NPs into the polymeric NCs can have two beneficial effects: on the one hand the obtained hybrid NCs can be directed particularly with the help of an external magnetic field towards the target, while on the other hand the application of an alternative external magnetic field can lead to the increase of the temperature in the tumor cells, inducing a hyperthermic effect that will destroy the tumor cells.

CS, a linear polysaccharide of randomly distributed N-acetyl glucosamine and glucosamine units, exhibits minimal foreign body reactions, controllable biodegradation properties, biocompatibility, hydrophilicity, non-toxicity, non-antigenicity, and antimicrobial activity. Moreover, it has been widely used for drug delivery and tissue engineering applications [19]. Compared to natural polymers, the synthetic polymers have high purity, good reproducibility, and can assure a long release time for the therapeutic agent [20]. In the present study, an alternating copolymer of itaconic anhydride with *N*-vinyl pyrrolidone was used as a synthetic copolymer. This copolymer is characterized by a high reactivity under mild temperature conditions due to the anhydride cycle, which is easily opened under the action of nucleophilic agents, such as the amine group of chitosan, without the presence of additional catalysts. The prepared NCs were loaded with 5-fluorouracil as the model drug, which is an anticancer drug with a broad spectrum that has been used for about 40 years to treat breast cancer, head and neck cancers, stomach cancer, colon cancer, and some skin cancers [21]. The method for the preparation of these types of hybrid NCs is simple, reproducible, uses non-toxic reagents, and has the advantage of increased drug encapsulation efficiency as compared to other types of NPs.

## 2. Results and Discussions

**Figure 1:**
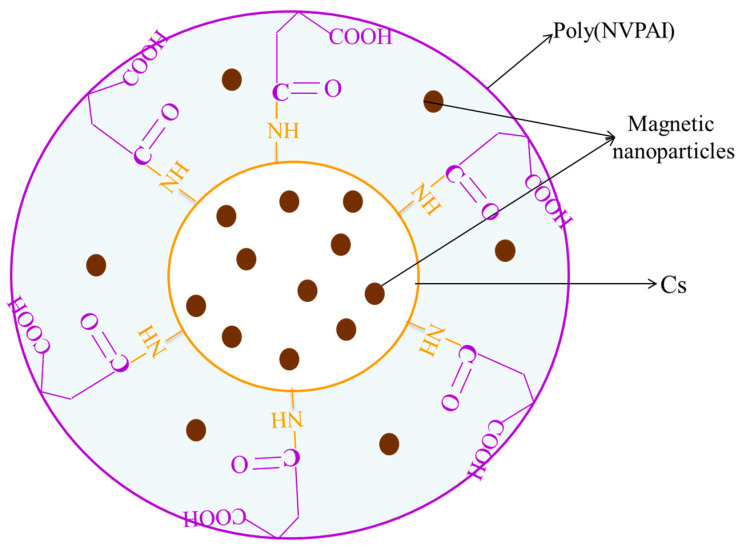
Schematic illustration of magnetic NCs based on CS and poly(NVPAI) containing magnetic nanoparticles.

One of the objectives of this study was to prepare the magnetic NCs via the incorporation of the magnetic nanoparticles into NCs based on chitosan (CS)/poly(N-vinyl pyrrolidone-*alt*-itaconic anhydride)-poly(NVPAI). These NCs are instantaneously formed at the interface between CS and highly reactive anhydride cycles of poly(NVPAI), resulting in a polymer membrane formed by the formation of amide bridges between the two polymers. A certain amount of anhydride cycles can hydrolyze at the interface with the aqueous CS solution, generating new carboxylic groups. The schematic chemical structure of the obtained magnetic NCs is shown in Figure 1. The presence of magnetic nanoparticles was noticed in both the core of NCs and in the polymer membrane, which has a hydrogel behavior.

### 2.1. FTIR Spectroscopy

**Figure 2:**
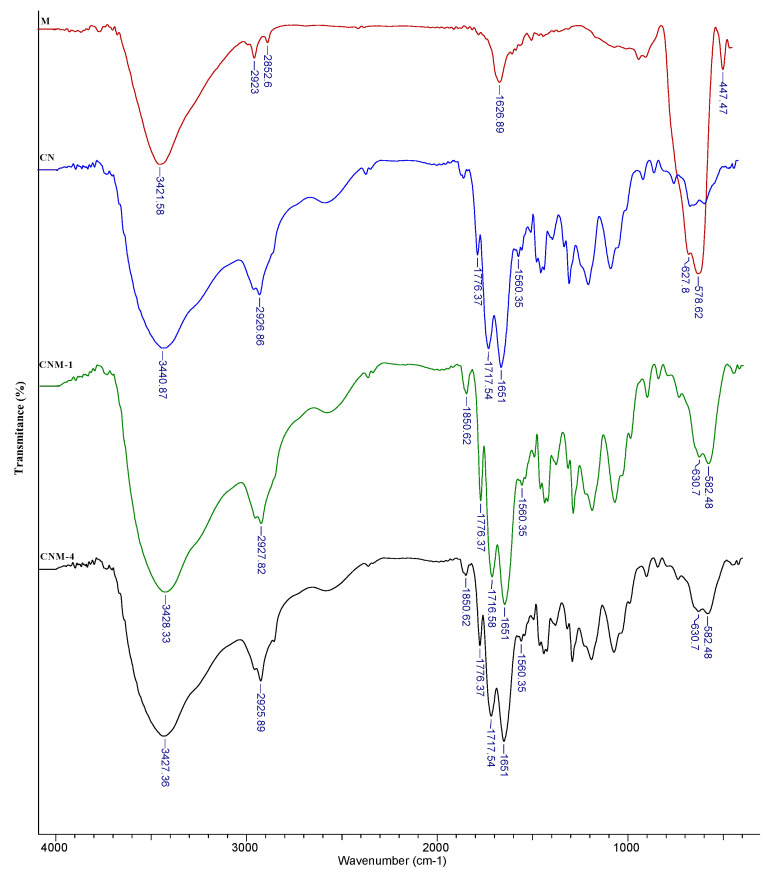
FTIR spectra for magnetite (M), CN, CNM-1, and CNM-4.

The structural characterization by Fourier-transform infrared spectroscopy (FTIR) of magnetic NPs (M), non-magnetic nanocapsules (CN) and magnetic NCs (CNM-1 and CNM-4) are presented in Figure 2. The FTIR spectra of M samples present absorption bands at 578 cm^−1^ and 627 cm^−1^ corresponding to the Fe-O-Fe bonds from magnetite (Fe_3_O_4_), [22,23], while the peaks at 1626 cm^−1^ and 3421 cm^−1^ are characteristic to the hydroxyl groups [24,25].

The similar characteristic peaks can be found for CNM-1 and CNM-4 samples. All of the absorption peaks from 3440 cm^−1^, 3426 cm^−1^, and 3427 cm^−1^ that appeared in the spectra of CN, CNM-1, and CNM-4 are attributed to the stretching vibration of the secondary amine group (N-H) or hydroxyl groups (O-H). Also, the CN, CNM-1, and CNM-4 samples show peaks at around 1776 cm^−1^ that can be attributed to the stretching vibration of the C=O group from the anhydride groups [1]. The absorption bands at 1717 cm^−1^ and 1716 cm^−1^ correspond to the stretching vibration of the –C=O from carboxylic groups, while the peak from 1650 cm^−1^ corresponds to the carbonyl bond from the amide groups. The presence of magnetic NPs in the composition of magnetic NCs (CNM-1 and CNM-4) was confirmed by the occurrence of the signal from 630 cm^−1^ and 582 cm^−1^ (Fe-O and Fe-O-Fe groups). However, a shift between the characteristic peaks of magnetite can be observed from 627 to 630 cm^−1^ and from 578 to 582 cm^−1^ after their incorporation into NCs, which may indicate the formation of ionic interactions between Fe_3_O_4_ and the polymeric matrix [26].

### 2.2. Mean Diameter Determination

**Figure 3:**
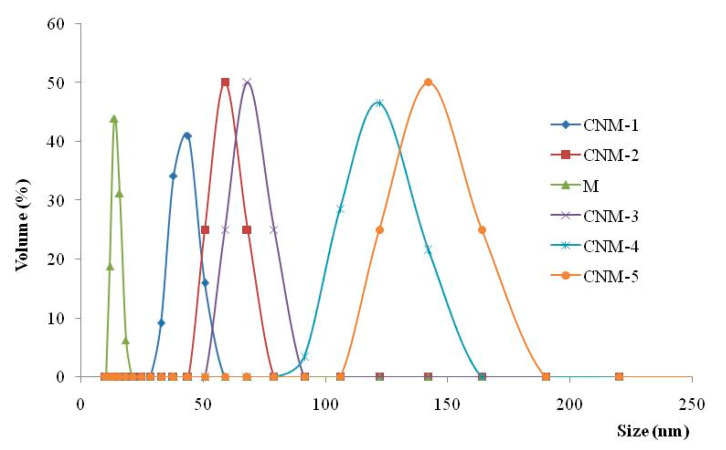
Dimensional distribution curves of magnetic nanoparticles (M) and hybrid nanocapsules (CNM).

The mean diameter of the magnetic NPs was around 14 nm, whereas the mean diameters for hybrid NCs varied between 43 and 142 nm (Figure 3).

From the obtained results, it was noticed that the size of magnetic NCs and the dimensional polydispersity increase with the increasing quantity of magnetic NPs. The amount of magnetite that was added to the CS solution is presented in Table 1.

This size increase can be attributed to the inclusion of magnetite in both the core of the NCs and in the polymer membrane. Granulometric distribution curves have a monomodal character but present broad polydispersity, especially when high amounts of magnetite were incorporated.

### 2.3. Aqueous Suspensions Stability

The zeta-potential of magnetic NCs was determined in order to investigate the stability of capsule dispersion in a slightly alkaline medium at pH 7.4, which mimics the physiological conditions. Zeta-potential values varied between −17.7 mV and −20.9 mV and increased with the increase of the amount of magnetite in the NCs (Table 2).

Additionally, it was found that increasing the ratio between the two phases (aqueous and organic) led to the increase of the zeta-potential values. The hydrolysis of a certain amount of the non-reacted anhydride cycles leads to the formation of carboxylic groups, which in alkaline medium form carboxylate anions. As a consequence, electrostatic repulsion forces are exercised between negative charged capsules, which lead to an increase in zeta-potential values and consequently to improved stability of the NC suspension and a reduced agglomeration tendency.

### 2.4. Nanocapsule Morphology

**Figure 4:**
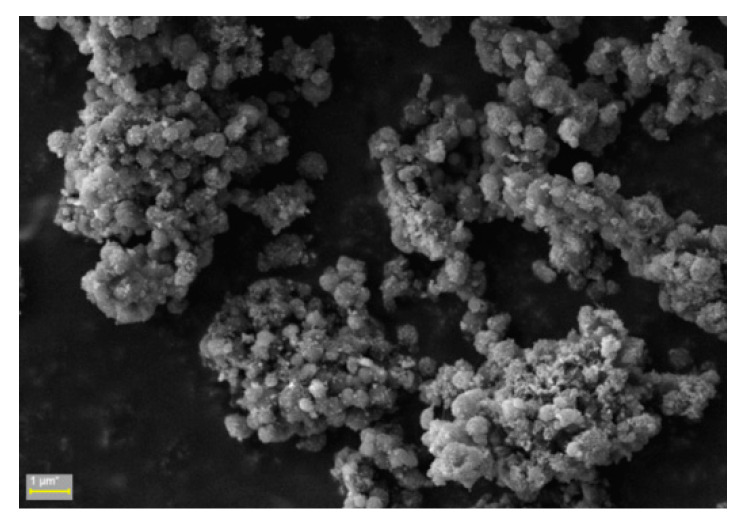
Scanning electron microscopy (SEM) for sample CNM-5.

The scanning electron microscopy (SEM) image of the CNM-5 sample is presented in Figure 4. From the figure, it can be observed that the NCs have a spherical shape. Moreover, the image shows the presence of a phase contrast on the surface of the magnetic NCs, which is direct proof of the incorporation of magnetic NPs, even on the surface of the NCs. Furthermore, the obtained hybrid NCs are suited for intravenous injections because their size is around a few hundred nanometers [27,28,29,30].

**Figure 5:**
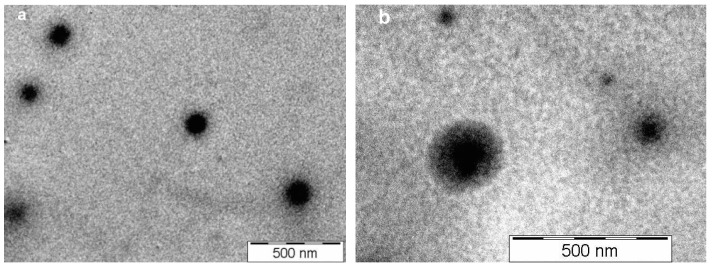
Transmission electron microscopy (TEM) for sample CNM-5 (**a**,**b**) with two different magnifications.

Based on the transmission electron microscopy (TEM) spectra in Figure 5, the capsules have a spherical shape (Figure 5a). Their “core–shell” structure is obvious, with their core being composed of agglomerated magnetite nanoparticles. This core is surrounded by the polymer membrane (the brighter halo around the capsule), but even in this membrane and partially outside of the membrane magnetite nanoparticles can be seen, as suggested by the previously presented SEM photograph. The photograph also allows the approximate estimation of the thickness of the polymer membrane (around 20 nm, according to Figure 5b).

### 2.5. Thermal Behavior

**Figure 6:**
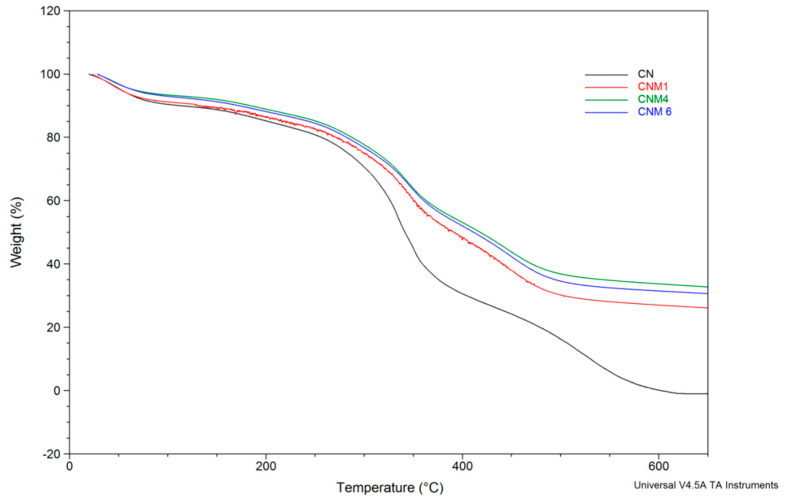
Thermogravimetric curves for CN, CNM-1, CNM-4, and CNM-6.

The characteristic thermogravimetric (TGA) curves for hybrid NC samples (CNM-1, CNM-4, and CNM-6) and non-magnetic NCs (CN) are presented in Figure 6.

From this figure it can be observed that all the thermal curves present three steps of degradation with different percentages of mass loss. The first step of degradation, which is attributed to the evaporation of free water molecules, appears in the temperature range of 25 °C to 120 °C and is characterized by a weight loss of between 8 and 10%. The second one, occurring in the temperature range of 120 °C to 400 °C and with a significant weight loss between 35 to 40%, corresponds to the breakdown of the anhydride rings of the itaconic anhydride units and to the thermal and oxidative decomposition of CS. The last step of degradation, observed between 400 °C and 650 °C, has a weight loss of approximately 25% and corresponds to the complete decomposition of the polymeric materials. There is a logical sequence for all the thermal curves. For example, for the CNM-1 sample a mass percentage of 26.9% was noticed, which remained at 700°C, corresponding to the magnetite amount that was incorporated in the hybrid NCs. Additionally, the percentage remaining in the CNM-4 sample was around 33.2%—this difference was linked to the initial magnetite/CS ratio incorporated into the NCs (Table 1). The CNM-6 sample, which had the same initial magnetite/CS ratio as CNM-4 sample, had a remaining mass percentage of 31%. This small difference between the CNM-4 and CNM-6 samples can be attributed to the change of the organic phase (when the organic phase increases, the amount of incorporated magnetite decreases) (Table 1). In the case of the non-magnetic NCs (CN), which were used as a reference, a complete decomposition at approximately 600 °C with a residue of 0% was noticed, because this material, which is based on organic polymers, cannot withstand temperatures over 600°C. These results, which are in complete agreement with other previous studies [31,32], showed that these magnetic NCs are thermally more stable than the non-magnetic NCs, and also that their thermal stability increases when the content of magnetite increases. The allure curves of magnetic NCs compared to non-magnetic NCs are the same, but there is a shift between magnetic and non-magnetic particles, which is due to the formation of ionic interactions between magnetite and the polymer matrix, as demonstrated by FTIR analysis, which led to the increased thermal stability of the magnetic NCs.

### 2.6. Magnetic Properties

**Figure 7:**
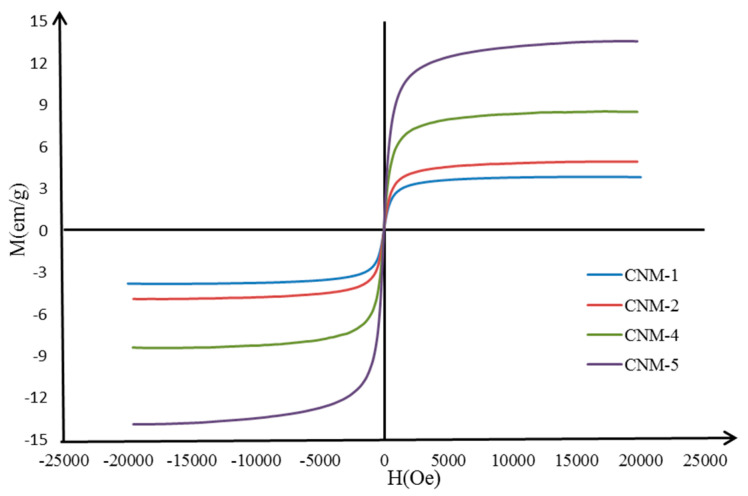
The magnetization curves of different magnetic NCs.

In Figure 7, the magnetization curves for the CNM-1, CNM-2, CNM-4, and CNM-5 samples are represented, which were determined through vibrating sample magnetometer (VSM) at room temperature. The magnetization curves do not exhibit hysteresis when an external magnetic field is applied for all the analyzed samples. The coercivity (Hc) and the remanence (Mr) are both zero, which proves the superparamagnetism of the magnetic NCs [33]. The concept is explained by the fact that when an external magnetic field is applied, the magnetite NPs becomes magnetic, returning to the non-magnetic state once the magnetic field is stopped (Supplementary Figure S1). This feature makes the application of the magnetic NCs possible for the targeted administration of drugs. The following saturation magnetization values (Ms) were recorded for each sample: Ms = 13.5 emu/g for the CNM-5 sample, Ms = 9.9 emu/g for the CNM-4 sample, Ms = 5.0 emu/g for the CNM-2 sample, and Ms = 4.0 emu/g for the CNM-1 sample.

The comparative analysis of the values of the saturation magnetization reinforces the idea that the magnetite NPs were successfully incorporated into the NCs. The highest magnetization value was recorded for the CNM-5 sample, which has the highest amount of magnetite, and it appears that the value of magnetization decreases when the amount of Fe_3_O_4_ decreases.

### 2.7. Swelling Degree of Magnetic Nanocapsules

**Figure 8:**
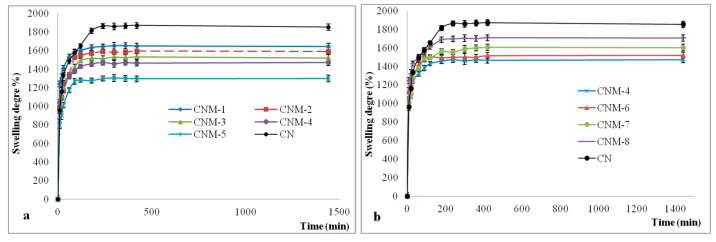
The swelling kinetics curves of the NCs in alkaline conditions (pH 7.4) for the following samples: (**a**) CN, CNM-1, CNM-2, CNM-3, CNM-4, CNM-5; (**b**) CN, CNM-4, CNM-6, CNM-7, CNM-8.

The swelling behavior of the obtained magnetic NCs was investigated in slightly alkaline medium (pH 7.4) in order to evaluate their potential to be used as drug carriers (Figure 8). The swelling of the NCs is caused by the water penetration within the empty core until complete filling, as well as by the swelling of the polymer membrane, which has a hydrogel character [34]. It is evident from Figure 8 that all the magnetic NCs presented a high swelling degree. Likewise, it can be observed that non-magnetic nanocapsules show a swelling degree of about 1850%. The much higher swelling degree in this case can be justified by the lack of the magnetic material in the nanocapsules’ composition. The size, composition, and preparation parameters of magnetic NCs have important impacts on the swelling properties. In the Figure 8a, it can be observed that the swelling degree of the magnetic nanocapsules varies between 1298% and 1640%, and decreases with the increase of the magnetite amount incorporated into the NCs.

This behavior can be attributed to the decrease of the space inside the NCs due to the increase of the amount of magnetite NPs present in the system, thus reducing the amount of water that can penetrate into the core of the capsule. In Figure 8b, an increase of the swelling degree with an increase of the volume of the organic phase for the same quantity of magnetite NPs can be noticed (Table 1). This behavior may be explained as follows: As the volume of the organic phase increases, the number of macromolecules of poly(NVPAI) that come into contact with a drop of the aqueous solution of CS is decreased progressively due to the dilution of the copolymer solution. As a result, the crosslinking density of the nanocapsule membrane decreases, and therefore the swelling rate increases.

### 2.8. Loading Efficiency of 5-Fluorouracil (5-FU)

This type of magnetic NC was developed in order to be used for the treatment of cancer. The encapsulating efficiency of 5-FU, which is used as a model antitumor drug, into magnetic NCs is presented in Table 3.

The drug quantity retained by the NCs depended on the magnetite/polymer ratio. The drug loading efficiency decreased with the increasing of amount of magnetite NPs; this effect was expected because the space left available to the drug in the core of the NCs was reduced by increasing the amount of magnetite NPs. It was also noticed that the loading efficiency increased with the increase of the volume of the organic phase. This effect is due to the reduction of the crosslinking density of the nanocapsule membrane, and therefore to the increase of its ability to include a higher number of drug molecules in the mesh of the network. This behavior is in concordance with the swelling degree of hybrid NCs in PBS medium at pH 7.4.

### 2.9. 5-FU Release from Magnetic Nanocapsules

**Figure 9:**
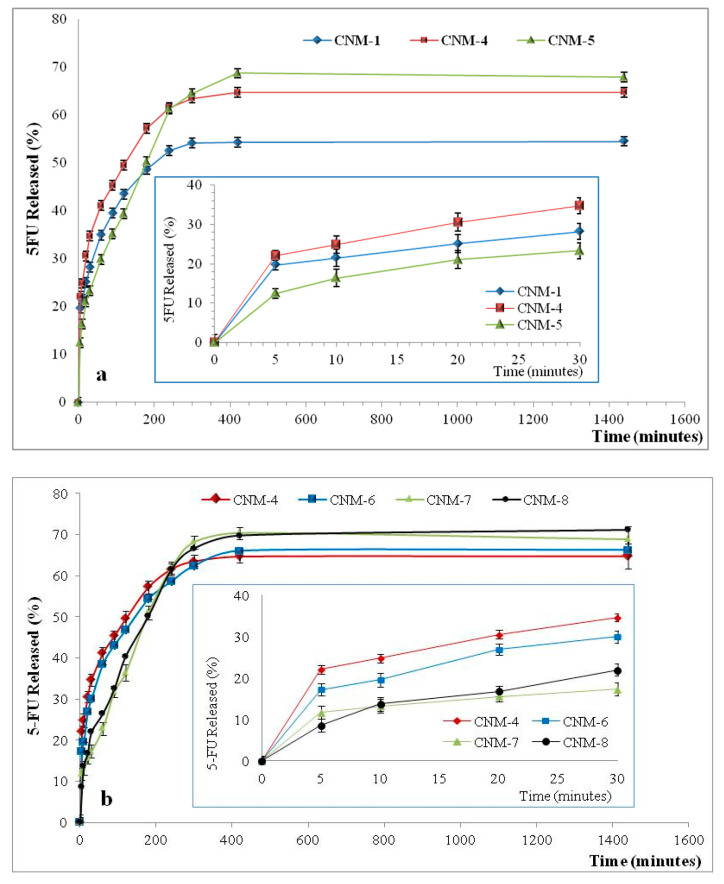
In vitro release kinetics of 5-FU from magnetic NCs in phosphate buffer solution (pH 7.4), with a zoom insert of release kinetics between 0 and 30 min for samples: (**a**) CNM-1, CNM-4 and CNM-5; (**b**) CNM-4, CNM-6, CNM-7 and CNM-8.

The kinetics of 5-FU release from the hybrid NCs in PBS (pH 7.4) are shown in Figure 9. The 5-FU release efficiency in alkaline medium was found to be between 52 and 70%.

The drug release from magnetic nanocapsules is controlled by diffusion of 5-fluorouracil and takes places in two steps. In the first phase, called the “burst effect”, a percentage of about 25–35% of 5-fluorouracil was released in the first 10–30 min, which can be attributed to the desorption of the drug situated on the surfaces of nanocapsules. The second phase corresponds to the diffusion of the drug from the inner core to the external phase. The evolution of the 5-fluorouracil release is almost linear, with a slow release rate until equilibrium [32]. Moreover, it seems that the increase of the amount of magnetic NPs led to an increase of the drug release efficiency (Figure 9a). Figure 9b shows that the release efficiency increases slightly with the increase of the volume of the organic phase. This behavior is in accordance with both the swelling degree and loading results, and is explained by the easier transport or diffusion of the drug through the polymeric membrane, which has a lower crosslinking density at high organic phase volume.

### 2.10. Theoretical Analysis of Drug Release

**Figure 10:**
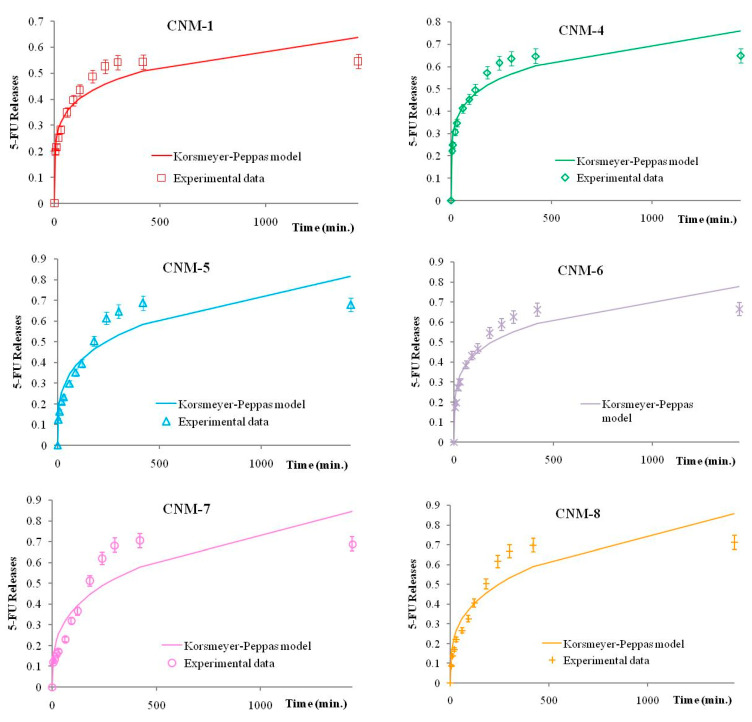
Experimental release kinetics and theoretical Korsmeyer–Peppas curves for magnetic nanocapsules.

The Korsmeyer–Peppas parameters values are presented in Table 4:

As one can see, in all cases the n values indicate a Fickian diffusion. Moreover, from the graphical representation (Figure 10) of the release kinetics and following the Korsmeyer–Peppas model, one can see that the best fit is in the first part of the release kinetics, i.e., in the swelling phase of the release, where the dominant phenomenon is the Fickian diffusion [35].

### 2.11. Viability Assay

**Figure 11:**
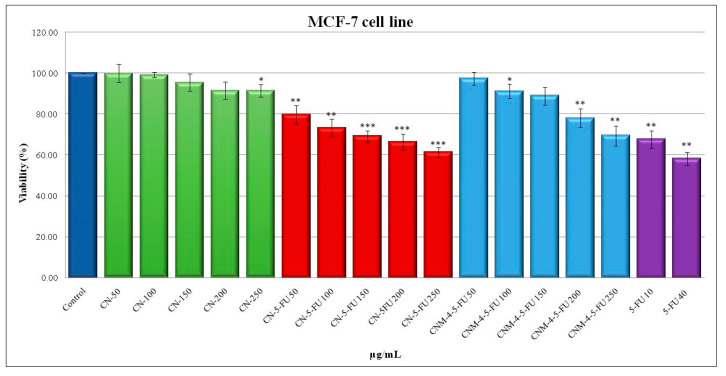
Viability of human breast carcinoma MCF-7 cells after 48 h incubation with non-loaded nanocapsules (CN-2), 5-FU-loaded nanocapsules (CN-2-5-FU), 5-FU-loaded magnetic nanocapsules (CNM-4-5-FU), and 5-FU as a function of concentration (Student’s *t* test: * *p* < 0.05; ** *p* < 0.01; *** *p* < 0.001).

The in vitro assays of the MCF-7 tumor cell line were performed in order to investigate the biocompatibility of the non-loaded and non-magnetic nanocapsules (CN) on the one hand, and on the other hand the cytostatic efficiency of 5-FU-loaded nanocapsules (CN-5-FU) and of 5-FU-loaded hybrid NCs (CNM-4-5-FU). This cytostatic effect was induced by the controlled release of 5-FU from the drug-loaded NCs during a treatment of 48 h at doses ranging from 50 to 250 µg/mL, and was compared to the effect of the treatment with free 5-FU. As shown in Figure 11, the 48-h treatment with the non-loaded nanocapsules (CN) resulted in insignificant interference with MCF-7 cell viability. Thus, compared to the control, at the lowest used dose (50 µg/mL) the cell viability reaches 99.84% and at the highest experimental dose (250 µg/mL) a value of 91.39% is reached, corresponding to cytotoxicity values of 0.16% and 8.61%, respectively. These values indicate that the analyzed samples are well tolerated by the MCF-7 cells. The treatment with 5-FU-loaded nanocapsules (CN-5-FU) was followed by a moderate decrease in cell viability in a dose-dependent manner.

At the minimum dose, a cell viability value of 79.7% was recorded, which correlated with a cytotoxic effect of 20.3%. At the maximum dose, the cell viability value was 61.33%, leading to a cytotoxicity of 38.67%. The CNM-4-5-FU treatment had a weaker cytotoxic effect than that induced by CN-5-FU, showing a value of 30.61% at the highest dose. The 48-h treatment with free 5-FU resulted in a decrease in the cell viability, reaching a value of 58.24% at the highest dose (40 µg/mL), which correlated with a cytotoxic effect of 41.76% and was close to the value recommended by in vitro screening programs [36,37,38].

## 3. Materials and Methods

### 3.1. Materials

The following materials were used in this study: chitosan (CS) (low molecular weight, 50,000–190,000 Da, degree of deacetylation 91%), acetone, dimethyl sulfoxide (DMSO), hexane, surfactants (Tween 80, Span 80), and 5-fluorouracil (5-FU) were purchased from Sigma Aldrich. Sodium hydroxide (NaOH), Pluronic F127, and iron (III) chloride anhydrous originated from Lachner. Iron(II) chloride tetrahydrate (FeCl_2_·4H_2_O) was purchased from Fluka. Poly(N-vinylpyrrolidone-*alt*-itaconic anhydride) poly(NVPAI) is an alternant copolymer that was synthesized in our laboratory by a radical copolymerization method [39]. Additionally, phosphate-buffered solution (PBS) at pH 7.4 and double-distilled water were produced in our laboratory. Breast carcinoma MCF-7 cell line was acquired from ATCC^®^HTB-22D™. Dulbecco’s modified growth medium (DMEM), penicillin, and streptomycin were purchased from Biochrom AG (Berlin, Germany), while fetal bovine serum (FBS) was acquired from Sigma-Aldrich (Berlin, Germany).

### 3.2. Magnetic Nanoparticle Preparation

Magnetic NPs can be synthesized by chemical, physical, or biological methods, but the chemical methods are most frequently used due to their simplicity, controllable handling, and efficiency [40]. In this context, the magnetite NPs were prepared by using the coprecipitation method described by Hritcu et al. [41], with slight modifications. The coprecipitation reaction in basic medium was carried out in a tightly closed flask under an inert atmosphere. Here, 0.0275 moles of iron (II) chloride tetrahydrate FeCl_2_·4H_2_O was dissolved in 84 mL of distilled water, while separately 0.055 moles of iron (III) chloride FeCl_3_ was dissolved in 90 mL of H_2_O. The two solutions were left under magnetic stirring until complete dissolution, and then in each of these solutions a further 36 mL of Pluronic F127 solution was added at a concentration of 2% as a non-ionic surfactant. Afterwards, the two solutions were poured into a well-closed 500 mL flask and placed into a heating bath (with a temperature of 70 °C) equipped with magnetic stirring. The obtained solution was maintained under bubbling of nitrogen to prevent premature oxidation of the iron (II) in iron (III). An aqueous sodium hydroxide solution (12.8 g in 120mL H_2_O) was added drop-by-drop in the flask and the coprecipitation reaction continued for 30 min. The precipitate was washed with double-distilled water to reduce the pH to 7 and to remove all traces of unreacted precursors, as well as all side products. The nanoparticles were recovered by the magnetic separation method using a strong magnet applied to the wall of the flask. The obtained precipitate was dried at a temperature of 50 °C in a vacuum for 24 h, and then kept in an oven at 50 °C until a constant mass of Fe_3_O_4_ was achieved.

### 3.3. Preparation of Magnetic Nanocapsules

Hybrid NCs based on CS, poly(NVPAI), and magnetite were obtained by the interfacial condensation method. The experiment begins with the preparation of both organic and aqueous solutions. The organic phase was prepared by dissolving 500 mg copolymer (poly(NVPAI)) in 15 mL of DMSO under magnetic stirring. After complete dissolution of the copolymer, 25 mL acetone and Span 80 (2%; *w/v*) were added under continuous stirring. When the influence of the volume ratio between the two phases (aqueous and organic) was studied, the quantity of poly(NVPAI) was kept constant and the volume of the organic phase was increased, keeping the same ratio between the volumes of DMSO and acetone. Separately, the aqueous phase was prepared by dissolving a specific amount of CS (150 mg) in 15 mL of 2% acetic acid solution at 65 °C under magnetic stirring, then the obtained solution was filtered and brought to room temperature. A certain volume of magnetite aqueous suspension (different amount of magnetite in 5 mL distilled water) was added by dropping into the CS solution under continuous stirring. The concentration of CS was calculated relative to the total volume of the aqueous phase (0.0075 g/mL). A non-ionic surfactant (Tween 80) with a concentration of 2% (*w/v*) was added to the formed solution of CS. The formed suspension was ultrasonicated and then slowly added drop-wise into the organic solution of poly(NVPAI) under continuous magnetic stirring at room temperature. After 2 h, the magnetic NC suspension was centrifuged for 15 min at 8000 rpm in order to separate NCs from the supernatant. Finally, these NCs were purified by repeated washings with double-distilled water, acetone, and hexane, then dried at room temperature at a constant weight. The considered variables were the amount of magnetite added to the CS solution and the volume ratio between aqueous and organic phases (Table 1).

The yield of the NCs was calculated according to the following equation:

(1) $$\mathrm{Nanocapsules}\;\mathrm{yield}\;\left(\%\right)=\frac{\mathrm{amount}\;\mathrm{of}\;\mathrm{recovered}\;\mathrm{nanocapsules}}{\mathrm{total}\;\mathrm{amount}\;\mathrm{of}\;\mathrm{polymers}\;\mathrm{used}+\mathrm{amount}\;\mathrm{of}\;\mathrm{magnetic}\;\mathrm{nanoparticles}}\times 100$$

### 3.4. Characterization

#### 3.4.1. Structural Characterization

Fourier transform infrared spectroscopy (FTIR) was used to confirm the formation of new amide groups and to demonstrate the presence of the magnetite in the prepared hybrid NCs. The structural characterization was performed with a Digilab Scimitar FTS 2000 FTIR spectrometer in the transmittance mode ranging from 400 to 4000 cm^−1^ using the pellet procedure with KBr.

#### 3.4.2. Morphological Characterization

Magnetic NCs were characterized from a morphological point of view (size, shape, and surface morphology) by SEM (Vega Tescan) and TEM. For TEM observation, a droplet of the capsule suspension in acetone was deposited onto a formvar-coated copper grid. The samples were analyzed with a Philips CM100 microscope equipped with an Olympus camera and transferred to a computer equipped with a Megaview system. The mean diameter, size distribution, and zeta-potential were determined in triplicate at 25 °C at a concentration of 1% (*w/v*). The mean diameters were determined by dynamic light scattering (DLS) (Zeta Nanosizer Malvern) on samples dispersed in anhydrous acetone in order to avoid the swelling of the NCs. A droplet of Span 80 was added in order to avoid aggregation of the NCs. The zeta-potential was determined by electrophoresis in phosphate-buffered solution (PBS; pH = 7.4).

#### 3.4.3. Thermal Properties

Thermogravimetric analyses (TGA) allowed the determination of sample weight loss as a function of temperature. These measurements were performed with a TA Instrument Q600 analyzer in air atmosphere (100 mL/min) at a heating rate of 10°C/min, from ambient temperature to 700°C. The samples weighed 8–10 mg, and in order to obtain comparable data the operation parameters were kept constant during all experiments.

#### 3.4.4. Magnetic Properties

The magnetic properties (the saturation magnetization—Ms; the remnant magnetization—Mr; coercivity—Hc) were evaluated by vibrating sample magnetometry (VSM) (MicroMag, Vibrating Sample Magnetometer, Model 3900, Princeton Measurements Corporation, Princeton, NJ, USA) on dried powder at room temperature.

#### 3.4.5. Swelling Behavior in Aqueous Solutions

The swelling degree of the magnetic NCs samples was analyzed in slightly alkaline aqueous medium at pH 7.4, which simulated physiological conditions using a gravimetric method in order to evaluate their behavior as potential drug carriers. It is well known that the drug loading and release capacity is influenced by the swelling degree, which is directly correlated with the crosslinking degree. A specific amount (30 mg) of dried NCs was weighed and immersed in an Eppendorf tube containing PBS. The obtained suspension was maintained at 37 °C ± 0.5 °C under magnetic stirring at 120 rpm for 24 h. At specific time intervals, the suspension was centrifugated, the supernatant was removed, the excess of liquid was removed by carefully blotting with filter paper, and the swollen sample was weighed. The NCs (dry and swollen) were weighed with an accuracy of ±0.0001 g on an electronic microbalance instrument. All experiments were performed in triplicate. The percentage of the swelling ratio (Q%) was determined with Equation (2):

(2) $$Q\left(\%\right)=\frac{W-W0}{W0}\times 100\;$$

where W is the weight of swollen sample (mg) and W0 is the initial weight of dry sample (mg).

#### 3.4.6. Drug Loading Studies

The drug loading process was carried out through a diffusion mechanism. In this study, 5-FU was used as a model drug. Briefly, 20 mg magnetic NCs were dispersed in 1.5 mL aqueous drug solution with a concentration of 10 mg/mL 5-FU. The suspension was maintained under magnetic stirring at 120 rpm at 37 °C ± 0.5 °C for 24 h, and then was separated by ultracentrifugation at 8000 rpm for 10 min. The amount of 5-FU loaded into NCs was calculated by the difference between the initial amount of 5-FU and the amount of 5-FU from the supernatant using a UV spectrometer (Nanodrop One, Thermo Scientific, Waltham, MA, USA) at 266 nm. The efficiency of 5-FU encapsulation (E_ef_ %) into NCs was calculated as follows:

ml = mi − ms(3)

(4) $$\mathrm{Eef}\;\%=\frac{\mathrm{mi}-\mathrm{ms}}{\mathrm{mi}}\times 100$$

where ml is the amount of loaded 5-FU (mg), mi is the initial amount of 5-FU (mg), and ms is the amount of 5-FU found in the supernatant (mg).

#### 3.4.7. Drug Release Kinetics

The in vitro drug release studies were realized by using the dialysis method. Each sample of 5-FU-loaded magnetic NCs was introduced into a dialysis membrane and was then individually immersed in flasks with 13 mL PBS at pH 7.4, a value that is similar to blood. This system was maintained at 37 °C ± 0.5 °C under continuous stirring at 120 rpm for the entire release period. At regular time intervals, 1 mL of solution was withdrawn and replaced with fresh PBS. The amount of drug released was quantified spectrophotometrically at a wavelength of 266 nm using a Nanodrop One instrument (Thermo Scientific). The efficiency of 5-FU release (Ref%) was calculated using Equation (5):

(5) $$\mathrm{Ref}\;\left(\%\right)=\frac{\mathrm{mr}}{\mathrm{ml}}\times 100$$

where mr is the amount of drug released from the magnetic nanocapsules (mg) and ml is the amount of the drug loaded into the nanocapsules (mg).

#### 3.4.8. Theoretical Analysis of Drug Release

The Korsmeyer–Peppas model is described by the below equation (Equation (6)):

(6) $$\frac{M_{t}}{M_{\infty }}=k\times t^{n}$$

where t is the release time; M_t_ is the amount of drug delivered at time t; M_∞_ is the total amount of drug delivered; k is a kinetic constant, which is a measure of the release rate; and n is the diffusional exponent, which gives an indication of the mechanism of the drug release. Various values are achieved, depending on the geometry of the release device: values up to 0.5 indicate a Fickian diffusion, 0.5–1.0 indicate anomalous (non-Fickian) transport (i.e., mixed diffusion and chain relaxation mechanisms), and 1.0 indicates case II transport (zero order); values of n greater than 1 reflect the so-called super case II transport.

#### 3.4.9. In Vitro Cytotoxicity Assay

##### Cell Culture

The compounds were tested on human breast carcinoma MCF-7 cell line (ATCC^®^HTB-22D™), maintained in Dulbecco’s modified growth medium (DMEM) supplemented with 10% fetal bovine serum (FBS), 100 IU/mL penicillin, and 100 µg/mL streptomycin at 37 °C in a humidified atmosphere of 5% CO_2_ in air.

##### Viability Assay

The effects of the tested NCs samples on cell viability were assessed by MTT assay, following a method described by Mosmann [42] and Laville et al. [43] and based on the capability of living cells to convert the water-soluble yellow substrate (3-(4, 5-dimethyl-2-thiazolyl)-2, 5-diphenyl-2H-teyrazolium bromide) into the insoluble purple formazan. The quantity of formazan is directly proportional to the number of living cells [44]. Summarily, the cells were routinely subcultivated twice a week by trypsin/EDTA procedure, then counted and incubated in 96-well plates (8 × 10^3^ cells/well) at 37 °C for 24 h. Different doses ranging from 50 µg/mL to 250 µg/mL of non-loaded and 5-FU-loaded magnetic NC suspensions were added and incubated with the MCF-7 cells for 48 h. The cells were also treated with 5-FU in corresponding doses with the lowest and highest doses used for the NCs. Subsequently, the cells were subjected to the MTT assay protocol by using the Biochrom EZ Read 400 microplate automatic reader at a wavelength of 570 nm. The cell viability was calculated using the following formula:

(7) $$\mathrm{Cell}\;\mathrm{viability}\;\left(\%\right)=\frac{\mathrm{Absorbance}\;\left(\mathrm{test}\right)}{\mathrm{Absorbance}\;\left(\mathrm{control}\right)}\times 100$$

### 3.5. Statistical Analysis

The in vitro experimental results are presented as means ± SE. The difference between the control group and the treated groups was analyzed by the Student’s test, with *p* < 0.05 being statistically significant [45].

## 4. Conclusions

The original approach proposed in this study was to develop an innovative hybrid system using nanocapsules based on natural and synthetic polymers, containing both magnetic nanoparticles and an antitumoral drug, in order to improve the efficiency of the antitumoral treatment. SEM and TEM analyses revealed that these magnetic NCs are in the nanometric size range and have a spherical morphology. Moreover, the analysis of the magnetic properties led to the conclusion that the particles exhibit superparamagnetism. Hybrid NCs allowed the encapsulation of an increased amount of 5-FU and presented a controlled drug release. Furthermore, it was demonstrated that these 5-FU-loaded magnetic NCs have good compatibility with the MCF-7 cell line and a cytostatic effect similar to that of free 5-FU after a treatment period of 48 h. These magnetic NCs could be directed with an external magnetic field towards the target and the movement of the magnetic nanoparticles led to the increase of the temperature in the tumor cells, inducing a hyperthermic effect. This effect, combined with the release of the antitumor drug (5-FU) to the tumor site in a controlled manner, can destroy tumor cells. The obtained results are encouraging and could be of real interest in the medical field for the treatment of breast cancer, since these preliminary in vitro studies will be the starting point for further in vivo testing.

## Figures and Tables

**Table 1:** Experimental program and codification of synthesized samples (CN- represent non-magnetic nanocapsules and CNM represent magnetic nanocapsules).

Sample Code	Molar Ratio (Moles of CS NH_2_Groups/Moles of Anhydride Cycles of Poly(NVPAI)	% CS (*w/v*)	Fe_3_O_4_/CS (%)	Aqueous Phase/Organic Phase Ratio (*v/v*)	Yield (%)
CN	0.3/1	0.75	-	1:2	45
CNM-1			20		38
CNM-2			30		40
CNM-3			40		44
CNM-4			50		53
CNM-5			80		57
CNM-6			50	1:2.5	68
CNM-7				1:3	78
CNM-8				1:3.5	81

**Table 2:** Zeta-potential values for magnetic NCs in PBS at pH 7.4.

Sample	Zeta-Potential (mV)	Conductivity (mS/cm)
CNM-1	−17.7	12.5
CNM-2	−18.5	14.1
CNM-3	−19.6	15.4
CNM-4	−19.8	18.7
CNM-5	−20.9	17.6
CNM-6	−17.9	18.2
CNM-7	−19.8	12.2
CNM-8	−20.1	16.9

**Table 3:** The5-FU encapsulation efficiency values.

Sample code	CNM-1	CNM-4	CNM-5	CNM-6	CNM-7	CNM-8
Loading efficiency (%)	29	26	22	27	29	32

**Table 4:** Korsmeyer–Peppas parameters.

Sample	CNM-1	CNM-4	CNM-5	CNM-6	CNM-7	CNM-8
k	0.167	0.195	0.112	0.157	0.090	0.094
n	0.184	0.187	0.273	0.220	0.308	0.303

## Supplementary Materials

Supplementary materials can be found at https://www.mdpi.com/1422-0067/21/16/5659/s1. Figure S1. The magnetic nanocapsules suspension in acetone, in absence (first image) and in presence (second and third images) of magnetic external field (produced by high force magnets).
