# Supplementary material for: Antitumoral Drug: Loaded Hybrid Nanocapsules Based on Chitosan with Potential Effects in Breast Cancer Therapy
Source: Int J Mol Sci. 2020 Aug 7;21(16):5659. doi: 10.3390/ijms21165659 (PMC7460861; doi:10.3390/ijms21165659)
Supplement: Supplementary file 1 [file ijms-21-05659-s001.pdf]

Supplementary material

Antitumoral Drug - Loaded Hybrid Nanocapsules Based on Chitosan with Potential Effect in Breast Cancer Therapy

Kheira ZANOUNE DELLALI [1,2,3], Delia Mihaela RATA [4*], Marcel POPA [3,4,5*], M'hamed DJENNAD [1], Abdallah OUAGUED [2] and Daniela GHERGHEL [6]

[1]University AbdelhamidIbn Badis of Mostaganem, Laboratory of Structure, Elaboration and Application of Molecular Materials , Mostaganem , Algeria

[2] University Hassiba Benboualiof Chlef, Faculty of Technology, Chlef BP 151 and Algeria

[3]Gheorghe Asachi Technical University of Iasi, Mangeron Bld. no. 73, 700050 Iasi and Romania

[4] "Apollonia" University of Iasi, Faculty of Medical Dentistry, Pacurari Street, No. 11, Iasi 700511 and Romania

[5] Academy of Romanian Scientists, Splaiul Independentei Street, No 54, 050094 Bucharest and Romania

[6]NIRDBS - Institute of Biological Research Iasi, Department of Experimental and Applied Biology, Lascar Catargi 47, Iasi, 700107, Romania

* *Corresponding authors:* Marcel POPA: Tel.: +40232278683, fax: +40232271311. Email address: marpopa2001@yahoo.fr; Delia Mihaela RATA: Tel.: +40-0232-210.310, fax: +40-0232-210.310. Email address: iureadeliamihaela@yahoo.com

Contents

The magnetic nanocapsules suspension in acetone, in absence (first image) and in presence (second and third images) of magnetic external field (produced by high force magnets)…………………....…………………………………………………………………………..**Figure S1**

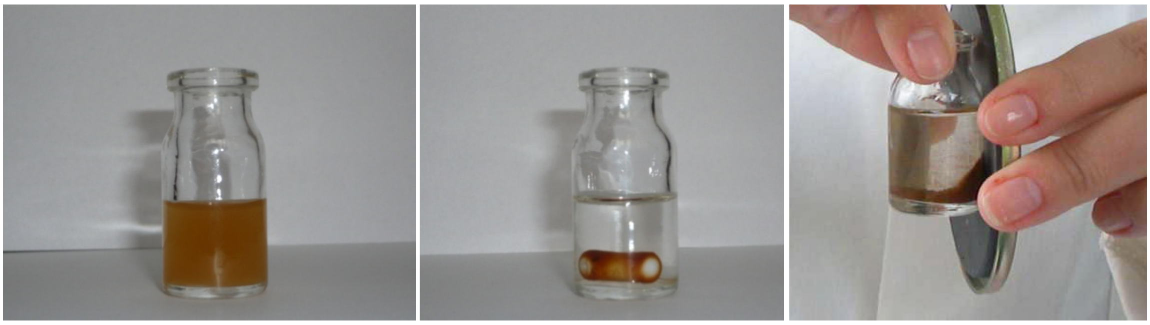

Figure S1. The magnetic nanocapsules suspension in acetone, in absence (first image) and in presence (second and third images) of magnetic external field (produced by high force magnets)

Acknowledgments: The authors would like to thank to **Prof. Dr. Leonard Ionut Atanase** from "Apollonia" University of Iasi, Faculty of Medical Dentistry, Pacurari Street, No. 11, Iasi 700511, Romania, for his assistance and interest in this study.
